# Peguero Electrocardiographic Left Ventricular Hypertrophy Criteria and Risk of Mortality

**DOI:** 10.3389/fcvm.2018.00075

**Published:** 2018-06-27

**Authors:** Hesham M. A. Afify, George S. Waits, Alia D. Ghoneum, Xiangkun Cao, Yabing Li, Elsayed Z. Soliman

**Affiliations:** ^1^Epidemiological Cardiology Research Center, Wake Forest School of Medicine, Winston-Salem, NC, United States; ^2^Department of Internal Medicine, Wake Forest School of Medicine, Winston Salem, NC, United States; ^3^Wake Forest School of Medicine, Winston Salem, NC, United States; ^4^Department of Internal Medicine, Section on Cardiology, Wake Forest School of Medicine, Winston Salem, NC, United States

**Keywords:** Peguero ECG-LVH criteria, prognostic performance, NHANES-III, electrocardiogram, ECG-LVH

## Abstract

**Background:** Peguero electrocardiographic left ventricular hypertrophy (ECG-LVH) criteria are newly developed criteria that have shown better diagnostic performance than the traditional Cornell-voltage and Sokolow-Lyon criteria. However, prediction of poor outcomes rather than detection of increased left ventricular mass is becoming the primary use for ECG-LVH criteria which requires investigating any new ECG-LVH criteria in terms of prediction.

**Aims:** To examine the prognostic significance of the newly developed Peguero ECG-LVH criteria.

**Methods:** We compared the prognostic significance of Peguero ECG-LVH with Cornell-voltage and Sokolow-Lyon ECG-LVH criteria in 7,825 participants (age 59.8 ± 13.4 years; 52.7% women) from the third National Health and Nutrition Examination Survey who were free of major intraventricular conduction defects. ECG-LVH criteria were derived from digital ECG tracings processed at a central core laboratory.

**Results:** At baseline, ECG-LVH was detected in 11.8% by Peguero; in 4.3% by Cornell voltage and in 6.4% by Sokolow-Lyon. During a median follow up of 13.8 years, 2,796 all-cause mortality events occurred. In multivariable models adjusted for demographics and cardiovascular risk factors, presence of Peguero ECG-LVH was associated with increased risk of all-cause mortality [HR (95% CI): 1.29 (1.16, 1.44)]. This association was not significantly different from the associations of Cornell voltage-LVH or Sokolow-Lyon LVH with all-cause mortality [HR (95%CI): 1.32 (1.12, 1.55) and 1.24 (1.07, 1.43), respectively; *p*-values for comparisons of these HRs with the HR of Peguero ECG-LVH 0.817 and 0.667, respectively]. Similar patterns of associations were observed with cardiovascular, ischemic heart disease and heart failure mortalities.

**Conclusion:** Peguero ECG-LVH is predictive of increased risk of death similar to the traditional ECG-LVH criteria.

## Introduction

Despite the low sensitivity of electrocardiogram (ECG) to detect left ventricular hypertrophy (LVH), ECG remains the most commonly used method for LVH screening owing to its low cost and wide availability (1). Development of electrocardiographic LVH (ECG-LVH) has been shown to be associated with increased risk of poor cardiovascular disease outcomes, and its regression reverses this risk (2). Interestingly, LVH detected by ECG has been shown to be predictive of poor outcomes as LVH detected by imaging (3–6). These findings along with its wide availability and low-cost have made the ECG the ideal tool for initial evaluation of patients with hypertension to detect LVH (7). Therefore, efforts to develop new ECG-LVH have continued to date. Recently, Peguero and colleagues proposed novel ECG-LVH criteria with a sensitivity reaching 62% to detect LVH by echocardiogram, which is much higher than the traditional ECG-LVH criteria (8). However, due to the better performance of ECG-LVH to predict poor outcomes more than its ability to detect anatomy (i.e., diagnose LVH), it has been suggested that risk stratification and prediction should be the primary use for ECG-LVH criteria (2). This has been underscored in the current ECG interpretation guidelines which recommend developing new ECG-LVH criteria for sole purpose of prediction (9). Therefore, the prognostic significance of Peguero ECG-LVH should be compared to the traditional ECG-LVH criteria before being utilized in clinical practice. Therefore, in the analysis from the United States third National Health and Nutrition Examination Survey (NHANES-III), we examined the prognostic significance of the Peguero ECG-LVH as a predictor for all-cause mortality, and compared the results to traditional ECG-LVH criteria; Cornell voltage LVH and Sokolow-Lyon LVH. In secondary analyses, we also investigated the associations with other causes of deaths including cardiovascular mortality, ischemic heart disease mortality, and heart failure mortality.

## Methods

NHANES III was designed to collect a nationally-representative population sample to estimate disease prevalence and the general health status of the United States. The NHANES-III survey was approved by the National Center for Health Statistics (NCHS) Research Ethics Review Board (ERB). Written informed consent was given by each participant upon enrollment, and the study conformed to the principles of the declaration of Helsinki. Between 1988 and 1994, initial home interviews were conducted to collect baseline information, including demographics (age, sex, race/ethnicity), medications data, past medical history, and behavioral data. Subsequently, participants visited mobile examination centers and gave blood samples to record basic laboratory values for each participant.

Diabetes was defined as a fasting plasma glucose ≥126 mg/dl (>7 mmol/L), hemoglobin A1c values ≥6.5%, or previous use of diabetes-related medications. Blood pressure was defined as the mean of three in-home measurements and three mobile center measurements using mercury sphygmomanometers. Hypertension was defined as an SBP ≥ 140 mmHg or DBP ≥ 90 mmHg. Dyslipidemia was defined as any of the following: total cholesterol >240 mg/dl (>6.21 mmol/L); low-density lipoprotein (LDL) cholesterol >160 mg/dl (>4.14 mmol/L); high-density lipoprotein (HDL) cholesterol ≤40 mg/dl (<1.03 mmol/L); or by the use of cholesterol-lowering medications. Body mass index (BMI) was calculated as the weight in kilograms divided by the height in meters squared. Obesity was defined as a BMI ≥ 30 kg/m^2^.

Standard 12-lead ECG was recorded on a Marquette MAC 12 system (Marquette Medical Systems, Milwaukee, Wisconsin) by trained technicians during the participant's visit to a mobile examination center, and the ECG data were automatically processed at a central core lab. Peguero ECG-LVH was calculated from the automatically measured waveforms. Perugia LVH was defined as deepest S wave in any single lead S_D_ + SV4 >2.3 mV for women and >2.8 mV for men (8). The sex-specific Cornell voltage and Sokolow-Lyon were used for comparison. ECG abnormalities were classified as major and minor using the Minnesota ECG classification (10). For this analysis, we only considered NHANES-III participants who underwent an ECG recording (*n* = 8,561). We excluded participants with poor quality ECGs or with major intraventricular conduction delay (i.e., complete bundle branch blocks and QRS duration ≥120 ms) or with missing mortality data, medical history, and anthropometric measurements.

Mortality data for NHANES III participants were available through December 31, 2006. A probabilistic matching algorithm based on 12 identifiers was used to link participants with death information captured in the National Death Index. Matching identifiers included social security number, gender, and date of birth. Follow-up was defined as the interval between the NHANES III examination and either of the following, depending on whichever came first: date of death, date of censoring, or December 31, 2006. The main end-point of all-cause mortality and secondary endpoints of cardiovascular disease, ischemic heart disease, and heart failure mortalities were examined and analyzed using data from the NHANES III Linked Mortality File. Participants who were unable to be matched with a death record were considered to be alive through the entire follow-up period.

Categorical variables were reported as frequency and percentage, while continuous variables were reported as mean ± standard deviation. Statistical significance for continuous variables was tested using the *T*-test while the chi-square method was used for the categorical variables.

The associations between Peguero ECG-LVH, Cornell voltage ECG-LVH and Sokolow-Lyon ECG-LVH with all-cause mortality were examined in separate Cox proportional hazards models. Models were adjusted for age, sex, race, hypertension, diabetes, dyslipidemia, obesity, current smoking, prior of coronary heart disease, prior of heart failure, serum creatinine, and major electrocardiographic abnormalities. In secondary analyses, the associations between Peguero ECG-LVH, Cornell voltage ECG-LVH, and Sokolow-Lyon ECG-LVH with cardiovascular mortality, ischemic heart disease mortality, and heart failure mortality, separately, were also examined. The hazard ratios associated with Peguero ECG-LVH, Cornell voltage ECG-LVH, and Sokolow-Lyon ECG-LVH were compared using the Chi-square calculated using the method described by Kaufman and MacLehose (11) and used in prior studies (12).

Data analyses were performed using SAS, version 9.4 (SAS Institute Inc., Cary, North Carolina). Statistical significance for all tests, including tests for interactions, was defined as *p* ≤ 0.05.

## Results

A total of 7,825 participants (52.7% women, and 49.5% non-Hispanic whites) were included in this analysis. At baseline, the age range was 40–90 years [mean 59.8 years (±13.4), median 60 years]. Baseline ECG-LVH was detected in 11.8% by Peguero; 4.3% by Cornell voltage and in 6.4% by Sokolow-Lyon. Table 1 shows the characteristics of the study participants stratified by ECG-LVH status. As shown, those with Peguero ECG-LVH were more likely to be slightly older, non-Hispanic black and with more prevalent cardiovascular risk factors such as current smoking, diabetes mellitus, hypertension, prior heart failure and prior coronary heart disease compared to those without Peguero ECG-LVH. Major ECG abnormalities and higher levels of serum creatinine were also more common in the study participants with Peguero ECG-LVH compared to those without Peguero ECG-LVH. On the other hand, obesity was more common in those without Peguero ECG-LVH, and no difference was observed between those with and without Peguero ECG-LVH regarding gender and dyslipidemia. Peguero ECG-LVH, Cornell voltage ECG-LVH and Sokolow-Lyon ECG LVH had showed similar distribution of most of the characteristics. However, differences existed in the association and level of significance with sex, diabetes, serum creatinine, and dyslipidemia (Table 1).

**Table 1:** Baseline characteristics of the study participants stratified by ECG-LVH status.

**Characteristics** ***Mean ± SD or n (%)***	**Peguero ECG-LVH**			**Cornell voltage ECG-LVH**			**Sokolow-Lyon ECG-LVH**		
	**Present** **(*n* = 923)**	**Absent** **(*n* = 6,902)**	***p-value***	**Present** **(*n* = 339)**	**Absent** **(*n* = 7,486)**	***p-value***	**Present** **(*n* = 498)**	**Absent** **(*n* = 7,327)**	***p-value***
Age (years)	60.9 ± 13.9	59.7 ± 13.4	0.01	66.9 ± 13.0	59.6 ± 13.4	<0.01	61.4 ± 13.8	59.7 ± 13.4	<0.01
Women	481 (52.1%)	3645 (52.8%)	0.70	274 (80.8%)	3852 (51.5%)	<0.01	216 (43.4%)	3910 (53.4%)	<0.001
Race/ethnicity			<0.01			<0.01			<0.01
Non-Hispanic white	391 (42.4%)	3,481 (50.4%)		127 (37.5%)	3,745 (50.0%)		162 (32.5%)	3,710 (50.6%)	
Non-Hispanic black	303 (32.8%)	1,531 (22.1%)		117 (34.5%)	1,717 (22.9%)		265 (53.2%)	1,569 (21.4%)	
Mexican-American	190 (20.6%)	1,607 (23.3%)		82 (24.2%)	1,715 (22.9%)		53 (10.6%)	1,744 (23.8%)	
Others	39 (4.2%)	283 (4.1%)		13 (3.8%)	309 (4.1%)		18 (3.6%)	304 (4.2%)	
Current Smoking	239 (25. 9%)	1,524 (22.1%)	0.01	39 (11.5%)	1724 (23.0%)	0.01	144 (28.9%)	1724 (22.1%)	0.01
Diabetes Mellitus	165 (17.9%)	745 (10.8%)	<0.01	80 (23.6%)	830 (11.1%)	<0.01	55 (11.0%)	855 (11.7%)	0.67
Hypertension	428 (46.4%)	2,400 (34.8%)	<0.01	209 (61.7%)	2,619 (35.0%)	<0.01	269 (54.0%)	2559 (34.9%)	<0.01
Prior coronary heart disease	80 (8.7%)	422 (6.1%)	<0.01	46 (13.6%)	456 (6.1%)	<0.01	43 (8.6%)	459 (6.3%)	0.036
Prior heart failure	72 (7.8%)	294 (4.3%)	<0.01	42 (12.4%)	324 (4.3%)	<0.01	31 (6.2%)	335 (4.6%)	0.091
Dyslipidemia	207 (22.4%)	1,718 (24. 9%)	0.10	72 (21.2%)	1853 (24. 7%)	0.14	95 (19.1%)	1,830 (25.0%)	0.003
Serum creatinine	1.2 ± 0.6	1.1 ± 0.4	0.04	1.2 ± 0.7	1.1 ± 0.4	0.12	1.2 ± 0.6	1.1 ± 0.4	<0.01
Obesity	142 (15.4%)	1,335 (19.3%)	<0.01	85 (25.1%)	1392 (18.6%)	<0.01	60 (12.1%)	1417 (19.3%)	<0.01
Major ECG abnormalities	229 (24.8%)	717 (10.4%)	<0.01	131 (38.6%)	815 (10.9%)	0.02	154 (30.9%)	792 (10.8%)	<0.01

*ECG-LVH, electrocardiographic left ventricular hypertrophy. Diabetes was defined as fasting plasma glucose ≥126 mg/dl (>7 mmol/L)„ hemoglobin A1c values ≥6.5%, or history of glucose-lowering medications. Hypertension was defined as systolic blood pressure > 140 mm Hg, diastolic blood pressure > 90 mm Hg or use of blood pressure lowering medications. Obesity was defined as body mass index ≥ 30 kg/m^2^. Dyslipidemia was defined as any of the following: total cholesterol > 240 mg/dl (>6.21 mmol/L); low-density lipoprotein (LDL) cholesterol > 160 mg/d (>4.14 mmol/L) l; high-density lipoprotein (HDL) cholesterol <40 mg/dl (<1.03 mmol/L); or use of cholesterol-lowering medications*.

**Figure 1:**
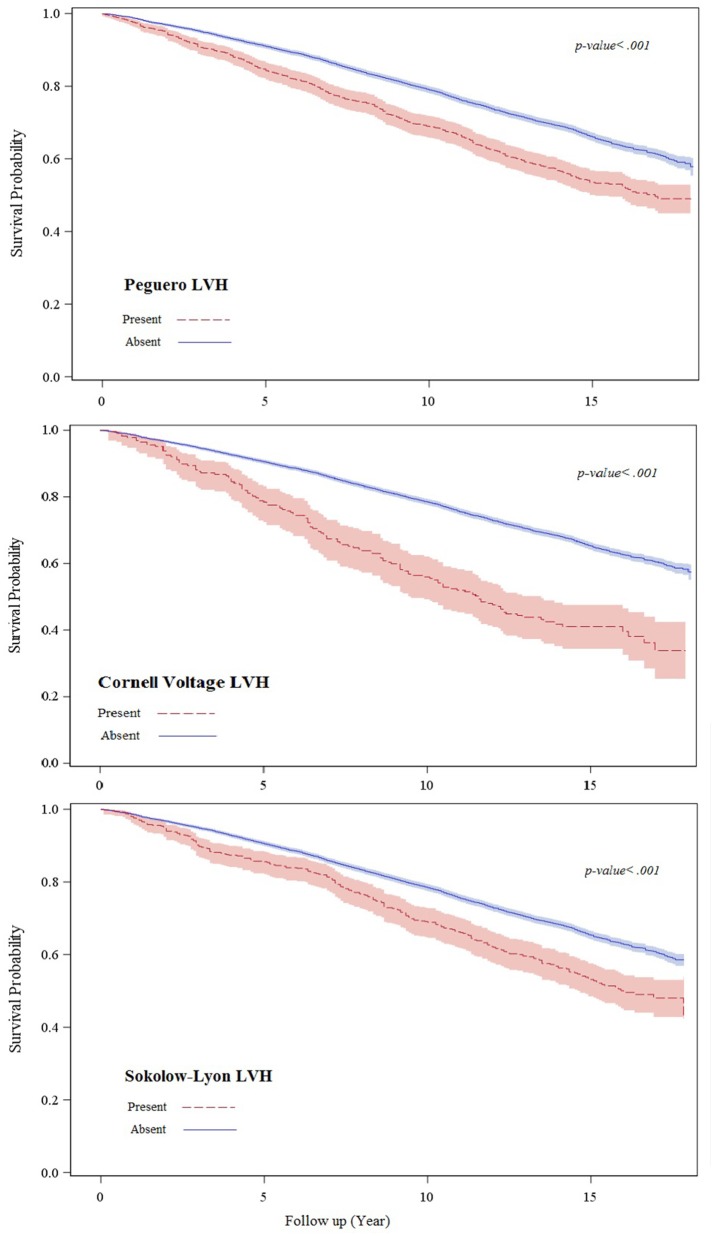
This figure shows the survival probability plot for all-cause mortality by ECG-LVH status.

During up to 18.1 years follow up (median 13.8 years) 2,796 all-cause mortality events occurred. More mortality events occurred during follow up in those with than those without baseline Peguero ECG-LVH (46.1 vs. 34.4%; *p* < 0.001) Cornell voltage ECG-LVH (55.5 vs. 34.8%; *p* < 0.01) or Sokolow-Lyon ECG-LVH (47.8 vs. 34.9%; *p* < 0.001). Figure 1 shows the survival probability plot for all-cause mortality by ECG-LVH status.

Among those who suffered all-cause mortality, 1,204 were identified as cardiovascular deaths, 689 as ischemic heart disease deaths, and 86 as heart failure deaths. Similar to what observed with all-cause mortality, more cause-specific deaths occurred during the 13.8 median follow up in those with than in those without baseline Peguero ECG-LVH (cardiovascular mortality: 23.5 vs. 14.3%; ischemic heart disease mortality: 12.6 vs. 8.3%; heart failure mortality: 2.3 vs. 0.9%) Cornell voltage ECG-LVH (cardiovascular mortality: 28.6 vs. 14.8%; ischemic heart disease mortality: 13.9 vs. 8.6%; heart failure mortality: 3.8 vs. 1.0%) and Sokolow-Lyon ECG-LVH (cardiovascular mortality: 23.3 vs. 14.9%; ischemic heart disease mortality: 11.7 vs. 8.6%; heart failure mortality: 2.6 vs. 1.0%).

In multivariable models adjusted for demographics and cardiovascular risk factors, Peguero ECG-LVH was associated with 29% increased risk of all-cause mortality. This association was not significantly different from the 32% increased risk of all-cause mortality observed with Cornell voltage ECG-LVH or the 24% observed with Sokolow-Lyon ECG-LVH (*p*-values for comparisons of these HRs with the HR of Peguero ECG-LVH 0.817 and 0.667, respectively) (Table 2).

**Table 2:** Peguero ECG-LVH and risk of all-cause mortality in comparison with traditional ECG-LVH criteria.

**ECG-LVH**	**Status**	**At risk/event**	**HR**	**HR**	**HR comparison**
		***N* (%)**	**(95% CI)**	***p-value***	***p-value***
Peguero	Absent	6,902 (34.4)	Reference		–
	Present	923/425(46.1)	1.29 (1.16, 1.44)	<0.001	
Cornell Voltage	Absent	7,486 (34.8)	Reference	–	0.817
	Present	339/188 (55.5)	1.32 (1.12, 1.55)	<0.001	
Sokolow-Lyon	Absent	7,327 (34.9)	Reference	–	0.667
	Present	498/238 (47.8)	1.24 (1.07, 1.43)	0.003	

*HR, Hazard ratio; CI, Confidence interval; ECG-LVH, electrocardiographic left ventricular hypertrophy. Model adjusted for age, sex, race, hypertension, diabetes, dyslipidemia, obesity, current smoking, prior coronary heart disease, prior heart failure, serum creatinine, and major electrocardiographic abnormalities*.

Peguero ECG-LVH also was associated with increased risk of cardiovascular, ischemic heart disease, and heart failure mortalities [HRs (95% CI): 1.29 (1.16, 1.44), 1.53 (1.31, 1.80), 1.40 (1.13, 1.73), and 2.35 (1.36, 4.06), respectively]. These associations were also not significantly different from the associations observed with Cornell voltage ECG-LVH [HRs (95%CI): 1.32 (1.12, 1.55), 1.39 (1.11, 1.75), 1.19 (0.86, 1.64), 3.04 (1.55, 5.95); p-values for comparisons of HRs with Peguero ECG-LVH = 0.817, 0.505, 0.407, 0.561, respectively] or Sokolow-Lyon ECG-LVH [HRs (95%CI): 1.24 (1.07, 1.43), 1.35 (1.10, 1.66), 0.09 (0.82, 1.46), 2.68 (1.41, 5.12); *p*-values for comparisons of HRs with Peguero ECG-LVH = 0.667, 0.351, 0.172, 0.762, respectively].

## Discussion

In this analysis from the NHANES-III we examined the prognostic significance of Peguero ECG-LVH, newly developed ECG-LVH criteria with superior diagnostic accuracy compared to traditional ECG-LVH criteria (8). Given the emerging trend of refocusing the use of the ECG-LVH criteria on the prediction of adverse outcomes instead of detection of increased left ventricular mass, we examined the associations of Peguero ECG-LVH with mortality. The key findings from our analysis are: (1) Peguero ECG-LVH is predictive of increased risk of all-cause and cause-specific mortalities; (2) Although the hazard ratios associated with Peguero ECG-LVH as a predictor for mortality were larger than that of Cornell voltage and Sokolow-Lyon ECG-LVH for some outcomes, none of them reached statistical significance. That is to say, the associations of Peguero ECG-LVH with all-cause and cause-specific mortalities were not different.

In a recent small patient-based population comprised of 138 patients with aortic stenosis, Peguero ECG-LVH was shown to be better than Cornell voltage and Sokolow-Lyon criteria regarding diagnosis of LVH as defined by echocardiography in all patients and by cardiac MRI in a subset of 41 patients (11). The same study showed that Peguero-LVH is associated with increased risk of all-cause mortality [HR (95%CI): 4.2 (1.1, 16.4)], which accord with our result. However, that study did not compare the risk of mortality associated with Peguero-LVH to that in Cornell voltage and Sokolow-Lyon, and the study did not examine causes of mortality. The small sample size and the patient-based population were just a few of the study limitations. (13).

In another patient-based population Peguero ECG-LVH was shown to be associated increased risk of cardiovascular death [HR (95% CI): 3.3 (2.5, 4.4)] similar to Cornell voltage [HR (95% CI): 3.3 (2.5, 4.3)] and slightly better than Sokolow-Lyon [HR (95% CI): 1.8 (1.5, 2.2)] (14). The similar risk associated with Peguero ECG-LVH and Cornell voltage accords with our findings. On the other hand, the difference between Peguero ECG-LVH Sokolow-Lyon was not formally tested in that study. Also, that study comprised of more than 91% men (generalizability issue) and the authors used a modified cut-off point for Peguero-LVH to fit their population which makes it hard to compare to our or other studies. Furthermore, the study did not include multiple modes of death as we did in our analysis.

Differences in the prognostic significance among ECG-LVH criteria have been reported (2). In the Multi-Ethnic Study of Atherosclerosis (MESA), there were wide variations in the associations of different ECG-LVH criteria with cardiovascular disease events, and the performance of these criteria varied by ethnicity (15). It even has been reported that the individual components of the same ECG-LVH criteria vary in their associations with outcomes. We have previously shown in the Atherosclerosis Risk in Communities (ARIC) study that the six components of Romhilt-Estes ECG-LVH criteria differ in their magnitude of association with different cardiovascular disease outcomes (16). Hence, it is not unexpected to see differences among ECG-LVH. The difference among ECG-LVH criteria may indicate that the ECG waveforms making these criteria may represent electrical biomarkers of different physiological phases within the myocardium.

LVH results in complex structural and functional remodeling of the myocardium which leads to altered ventricular conduction (17). It has been shown that increase in left ventricular mass is not the only determinant of QRS voltage which is a crucial ECG waveform involved in almost all ECG-LVH criteria (18). This provides further support that the ECG criteria for LVH do not necessarily mirror changes in LV mass all the time, which explains the too many imperfect ECG LVH criteria and their discrepancies in the detection of LVH (19). This may explain why the better diagnostic performance of ECG-LVH criteria such as Peguero is not necessarily paralleled by equal predictive performance when compared to the same ECG-LVH criteria.

One of the essential applications of ECG-LVH, given the wide availability of ECG, is its potential utility in the assessment of hypertension therapy. It is already established that successful management of high blood pressure improves LVH (20, 21). It is also known that LVH is associated with increased risk of poor outcomes, and regression of LVH reveres this risk (22, 23). Therefore, ECG criteria such as Peguero ECG-LVH could be useful in this context with its high diagnostic ability reported before (8) and the prognostic significance we report here. Nevertheless, further research may be warranted to confirm the superiority of Peguero ECG-LVH regarding diagnostic performance using a gold standard for detecting left ventricular mass such as cardiac MRI and also to confirm superiority regarding prognostic significance using other outcomes.

Our study should be read in the context of particular limitations. We only compared Peguero ECG-LVH to Cornell voltage and Sokolow-Lyon despite the fact that there are many other ECG-LVH criteria (9). However, we decided to focus on those two criteria for couple reasons: First; Peguero et al. used Cornell voltage and Sokolow-Lyon ECG- LVH criteria to compare the diagnostic performance of their newly developed criteria (8), and hence using them in our analysis as well completes the comparison form all aspects regarding diagnosis and prognosis. Second, Cornell voltage and Sokolow-Lyon ECG-LVH criteria are among the most commonly used criteria, and the current ECG interpretation recommendations do not favor one set of ECG-LVH criteria over the other (9). Therefore, it is appropriate to focus on Cornell voltage and Sokolow-Lyon ECG-LVH criteria as it would be the case for any other criteria. Other limitations inherent to all similar types of research include residual confounding despite adjusting for several potential confounders as well as issues with generalizability to populations not included in the analysis. Despite these limitations, this is the first study in a community-based population to evaluate the prognostic significance of newly emerging ECG-LVH criteria that could have potential in the assessment of monitoring the successful management of hypertension.

## Conclusions

In this analysis from the NHANES survey we showed that Peguero ECG-LVH is associated increased risk of mortality. This suggests its potential usefulness not only in detection of increased LV mass (LVH) but also in predicting cardiovascular events.

## Author contributions

ES: study concept and design; HA: drafting of the manuscript; ES, GW, AG, XC, and YL: critical revision of the manuscript for important intellectual content; ES, YL, HA: analysis and interpretation of data.

### Conflict of interest statement

The authors declare that the research was conducted in the absence of any commercial or financial relationships that could be construed as a potential conflict of interest.
